# Cytochrome c Negatively Regulates NLRP3 Inflammasomes

**DOI:** 10.1371/journal.pone.0167636

**Published:** 2016-12-28

**Authors:** Chong-Shan Shi, John H. Kehrl

**Affiliations:** B Cell Molecular Immunology Section, Laboratory of Immunoregulation, National Institute of Allergy and Infectious Diseases, National Institutes of Health, Bethesda, Maryland, United States of America; Tokyo Daigaku, JAPAN

## Abstract

The release of cytochrome c from the inner mitochondrial membrane, where it is anchored by caridolipin, triggers the formation of the Apaf-1 apoptosome. Cardiolipin also interacts with NLRP3 recruiting NLRP3 to mitochondria and facilitating inflammasome assembly. In this study we investigated whether cytosolic cytochrome c impacts NLRP3 inflammasome activation in macrophages. We report that cytochrome c binds to the LRR domain of NLRP3 and that cytochrome c reduces the interactions between NLRP3 and cardiolipin and between NLRP3 and NEK7, a recently recognized component of the NLRP3 inflammasome needed for NLRP3 oligomerization. Protein transduction of cytochrome c impairs NLRP3 inflammasome activation, while partially silencing cytochrome c expression enhances it. The addition of cytochrome c to an *in vitro* inflammasome assay severely limited caspase-1 activation. We propose that there is a crosstalk between the NLRP3 inflammasome and apoptosome pathways mediated by cytochrome c, whose release during apoptosis acts to limit NLRP3 inflammasome activation.

## Introduction

Apoptosis is an active, programmed process of autonomous cellular death. Signals that trigger apoptosis lead to the assembly of the apoptosome, a cytosolic protein complex that uses Apaf-1 as a sensor to detect cytosolic cytochrome c released from stressed or damaged mitochondria [1]. Upon oligomeration Apaf-1 recruits and activates caspase-9 and in-turn caspase-3, leading to apoptosis [2]. Apoptosis is considered a non-inflammatory form of cell death. Similar to the apoptosome, an inflammasome is a cytosolic protein complex that activates a caspase. NLRP3 (NLR family, pyrin domain containing 3) inflammasomes use NLRP3 as a sensor protein, which in the course of inflammasome activation recruits the adaptor Asc and caspase-1 resulting in caspase activation [3]. The activated caspase cleaves pro-IL-1β and pro-IL-18 releasing the mature cytokines and in some instances causing pyroptosis, an inflammatory form of cell death [4].

NLRP3 belongs to the nucleotide-binding domain and leucine rich repeat (LRR) containing NLR protein family [3]. Activation of NLRP3 inflammasomes in macrophages requires two signals, an initial priming signal needed to activate pattern recognition receptors, such as TLR4 and NOD2, or cytokine receptors, such as TNFR and IL-1R, which leads to NF-κB activation. The activated NF-κB increases the transcription and eventual translation of NLRP3 and pro-IL-1β [3]. The initial priming signal occurs rapidly and also involves a variety of post-translational modifications including changes in NLRP3 and ASC ubiquitination and ASC phosphorylation [5, 6]. Despite many studies the precise nature of the second signal needed for NLRP3 inflammasome activation remains elusive although cation flux, ER stress, and mitochondrial dysfunction have each been implicated as being either necessary or sufficient [3]. Recently the protein kinase NEK7 was found to bind the LRR domain of NLRP3 and to regulate its oligomerization and the activation of the inflammasome [7–9]. It functioned downstream of potassium efflux in NLRP3 activation. However, the catalytic activity of NEK7 was dispensable.

A resident inner mitochondrial membrane diphospholipid, cardiolipin, was also shown to interact with the LRR domains of NLRP3. Furthermore, its depletion by inhibiting its synthesis limited NLRP3 inflammasome activation [10]. Cardiolipin is best known for its role in mitochondrial function and apoptosis [11]. During intrinsic apoptosis cytochrome c oxidizes cardiolipin, which releases cytochrome c into the cytosol helping to trigger apoptosome formation. It also plays a critical role in the activation of caspase-8 and the final downstream activation of caspase-3 in the extrinsic pathway of apoptosis [12]. The shared usage of cardiolipin by the apoptosis and inflammasome pathways and overall similarity of the two pathways has been previously noted [3]. The known release of cytochrome c during NLRP3 inflammasome activation [13, 14] prompted us to investigate whether cytochrome c might impact inflammasome activity. We found that cytochrome c release reduced NLRP3 inflammasome activation and that it likely does so by limiting the association of NLRP3 with both cardiolipin and NEK7.

## Material and Methods

### Reagents and antibodies

Antibodies used for immunoblot analysis were the following: anti-NLRP3, anti-IL-1β, anti-caspase-1, anti-caspase-9 and anti-cytochrome c (Cell Signaling); and anti-Myc (Clontech Laboratories), anti-Flag (Sigma-Aldrich), anti-HA (Cell Signaling), anti-ASC (Santa Cruz Biotechnology), anti-actin conjugated to horseradish peroxidase (Sigma-Aldrich), goat anti-rabbit HRP-linked antibody, and horse anti-mouse HRP-linked antibody (Cell Signaling). Purified cytochrome c protein (Sigma-Aldrich), Profect-P1-lipid based protein delivery reagent (Targeting Systems), cardiolipin beads (Echelon), and the mitochondrial fractionation kit (Active Motif) were purchased and used as directed by the manufactures. The LPS, ATP, and Poly (dA-dT) were purchased from Sigma-Aldrich.

### Cells, plasmids and siRNAs

THP-1 and HEK 293T cells were obtained from the American Type Culture Collection. THP-1 cells were maintained in RPMI 1640 Medium supplemented with 10% FBS (Invitrogen), and HEK 293T cells were maintained in DMEM Medium with 10% FBS. To differentiate THP-1 cells into macrophages the cells were treated with 25 ng/ml of PMA (Sigma-Aldrich) for 3 hours. Subsequently, the cells were washed with Opti-MEM media (Life Technoligies) and re-seeded into 12 well plates in 0.5 ml of Opi-MEM media. The Myc tagged NLRP3 plasmids were a kind gift from Dr. Yong-Jun Liu (Baylor Institute for Immunology Research). The HA-NEK7 plasmid were a gift from Dr. Gabriel Nunez (University of Michigan). Cytochrome c and scrambled control siRNAs were purchased from Santa Cruz Biotechnology. The plasmids or the siRNAs were transiently transfected into the cells using X-tremeGENE-HP (Roche) following the manufacture’s protocol. Cytochrome c protein was delivered into THP-1 cells using Profect-P1 (Targeting systems) following the manufacture’s protocol.

### Immunoblot analysis and immunoprecipitations

For standard immunoblotting, the cells were lysed in the lysis buffer of 20 mM HEPES, pH 7.4, 50 mM β-glycerophosphate, 1 mM Na3VO4, 0.5% (vol/vol) Triton X-100, 0.5% (vol/vol) CHAPS (3-[(3-cholamidopropyl)-dimethylammonio]-1-propane sulfonate hydrate) and 10% (vol/vol) glycerol with a protease inhibitor 'cocktail' tablet (Roche). The lysates were separated by SDS-PAGE and transferred to nitrocellulose membrane by iBLOT Gel Transfer System (Invitrogen). The membrane was incubated with 5% nonfat milk w/v in TBS buffer (25 mM Tris-HCl, pH 7.5; 150 mM NaCl; 0.1% Tween-20) for 1 h, and then reacted with the primary antibody in TBS buffer with 2.5% nonfat milk or 5% BSA w/v overnight by shaking at 4°C. The appropriate second antibodies conjugated to HRP were used to detect the protein of interest via enhanced chemoluminescence (ECL). To immunoprecipitate endogenous or tagged proteins, the cells were lysed in the above buffer and incubated for 2 h at 4°C with the appropriate antibodies conjugated beads or primary antibodies followed by protein G beads for another 1 h. The captured imunoprecipitates were washed eight times with lysis buffer, then separated by SDS-PAGE, and analyzed by immunoblotting. For the cytochrome c protein binding assay, purified cytochrome c protein (50 ng) was added to immunoprecipitated Myc-tagged NLRP3 proteins for an additional 30 min. The beads were washed, the bound proteins eluted in SDS-sample buffer, size fractionated by SDS-PAGE, and analyzed by immunoblotting. The plasmids expressing Myc-NLRP3 and its truncated versions were transfected (200 ng) into HEK 293T cells overnight. To check whether purified cytochrome c protein affected the binding of NEK7 to NLRP3, plasmids expressing NEK7 (100 ng) and NLRP3 (100 ng) were separately transfected into HEK 293T cells. The NEK7 containing cell lysate was added to NLRP3 immunoprecipitates in the presence of either purified cytochrome c (100 ng) or BSA. The samples were incubated for 1 h and washed prior to immunoblot analysis. The western blotting results were quantitated using ImageJ.

### Cardiolipin pull down assay

HEK 293T cells were transfected with NLRP3-Flag construct (300 ng) overnight, then the cells were lysed with the above mentioned lysis buffer. The lysate was divide into 2 equal fractions. BSA or purified cytochrome c protein (50 ng) was added into the lysates, and the lysates incubated for 30 minutes with a gentle rotation at 4°C. Afterwards cardiolipin-coated agarose beads (Echelon) were added for an additional 30 minutes to pull down cardiolipin binding proteins. The beads were washed 8 times with the lysis buffer, the proteins eluted, and processed for immunoblotting.

### Inflammasome activation assay

To assess NLRP3 inflammasome activation THP-1 cells were treated with PMA 25 ng/ml for 3 h and the cells washed once with Opi-MEM medium (Life Technologies). The cells were reseeded with 0.5ml Opi-MEM medium in 12 well plate. LPS (50 ng/ml) was used to prime the cells overnight, and ATP (5 mM) was added for 1 h the following day after which the cell supernatants were collected. To assess AIM2 inflammasome activation PMA treated and LPS primed (2 h) THP-1 cells were transfected with 2 μg of Poly(dA-dT) and cell supernatants collected 6 h later. Following the culture period the supernatants were transferred to a microcentrifuge tube and 0.5 ml of methanol and 0.125 ml chloroform added. After vortexing and a 5 minute spin at 13,000 rpm the upper phase was discarded being careful not to disturb the interface. 0.5 ml methanol was added the samples spun again for 5 minutes at 13,000 rpm. The supernatants were discarded, and the pelleted proteins air dried for 5 minutes at 50°C. After which 60 μl of 1x sample loading buffer with DTT (final concentration of 0.1 M) was added to each sample prior to SDS-PAGE and immunoblotting to detect IL-1β and caspase-1 p20.

### *In vitro* caspase-1 activation assay

HEK 293T cells were treated with ATP 5 mM for 1 h, and non ATP treated cells served as a control. The mitochondrial fractions from these cell lysates were used as the trigger for NLRP3 inflammasome activation. The ATP-treated or non-treated mitochondrial fractions were mixed with cytosolic fractions from LPS-primed THP-1 cells. The samples were incubated at 30°C for 30 minutes in the presence of BSA (50 ng) or cytochrome c (50 ng). The samples were immunblotted to detect active caspase-1. The cellular fractions were performed following the manufacture’s protocol (Active Motif). To detect whether purified cytochrome c protein reduced NEK7 induced NLRP3 inflammasome activation NLRP3 (20 ng), ASC (10 ng) and caspase-1 (100 ng) were co-expressed, and NEK7 (100 ng) was expressed separately in HEK 293T cells. Their lysates were mixed with purified cytochrome c protein (50 ng) or BSA (50 ng), and then incubated 30°C for 30 minutes. Immunoblot analysis was used to detect caspase-1 p20.

### Cytochrome c mediated caspase 9 activation

Cell lysates were prepared from LPS stimulated differentiated THP-1 cells and HEK 293T cell expressing HA-NEK7 or not. The cell lysates were mixed and incubated with or without cytochrome c (0.25 mg/ml) for 30 minutes at 30°C. The status of full length and cleaved caspase-9 was assessed by immunoblotting.

### Statistics

All experiments were repeated a minimum of three times unless otherwise indicated. Statistical significance is based on the analysis of at least triplicate samples. Standard errors of the mean (SEM) and *p* values were calculated using *t* test in GraphPad Prism (GraphPad software).

## Results and Discussion

### Cytosolic cytochrome c release accompanies NLRP3 inflammasome activation

**Fig 1:**
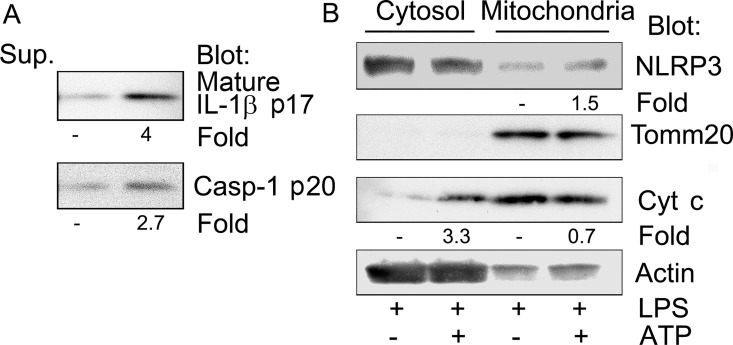
ATP treatment induces cytochrome c release. PMA differentiated THP-1 cells were primed with LPS 50 ng/ml overnight. One hour before harvesting the cells 5 mM ATP was added. Cell lysates were fractionated into a cytosolic and mitochondria enriched fractions. (A) Immunoblots of IL-1β and caspase-1 p20 present in the collected cell supernatants to verify NLRP3 inflammasome activation. (B) Immunoblots of cytosol and mitochondria enriched fractions from the above cells to assess cytochrome c release following NLRP3 inflammasome activation.

We first sought to verify reports that NLRP3 inflammasome activation is accompanied by a release of cytochrome c [13, 14]. To do so we isolated cytosolic and mitochondrial fractions from THP-1 cells previously primed with LPS and treated with ATP to activate inflammasome assembly. We checked that our LPS priming and ATP treatment were effective by immunoblotting cell culture supernatants for the presence of mature IL-1β and activated caspase-1 (Fig 1A). We fractionated the cells into mitochondria and cytosol enriched fractions and immunoblotted for NLRP3; cytochrome c; Tomm20, a resident mitochondrial protein [15]; and actin for a loading control. NLRP3 was present in both the cytosolic fraction and mitochondrial fraction and ATP treatment resulted in a small increase in NLRP3 in the mitochondria fraction. When normalized to Tomm20 levels, the ATP treatment resulted in a 1.5 fold increase in NLRP3 in the mitochondria fraction and when normalized to actin, a 3.3 fold increase in cytochrome c in the cytosolic fraction (Fig 1B). These data indicate that ATP treatment induced NLRP3 inflammasome activation and that some mitochondrial cytochrome c also can be released into the cytosol.

### Cytochrome c interacts with NLRP3 reducing the NLRP3/cardiolipin interaction

**Fig 2:**
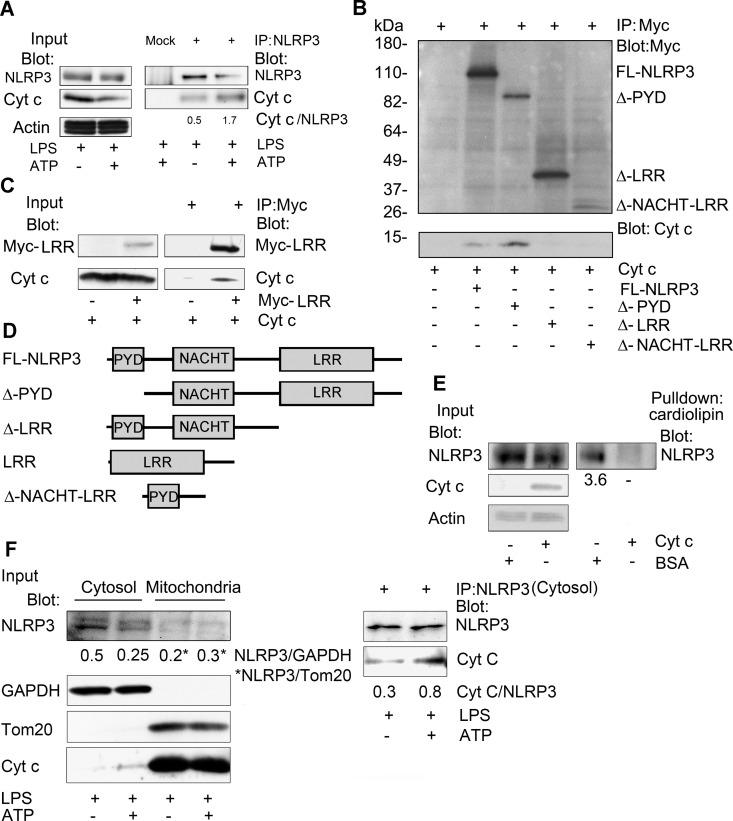
Cytochrome c binds to the LRR repeats of NLRP3 and interferes with cardiolipin binding. (A) Immunoblots of cell lysates and either NLRP3 or sham immunoprecipitates prepared from LPS-primed THP-1 macrophage cells, stimulated with ATP or not, to detect an interaction between endogenous NLRP3 and cytochrome c. The ratios between the NLRP3 and cytochrome c levels in the immunoprecipitates were quantitated using Image J. (B) Immunoblots of cell lysates and either control or Myc immunoprecipitates to map the portion of NLRP3 important for the interaction with cytochrome c. Myc-tagged NLRP3 constructs were transfected into HEK 293T cells. The immunoprecipitates were washed, incubated with cytochrome c (50 ng), washed again, fractionated by SDS-PAGE, and immunoblotted. (C) Immunoblots of cell lysates and Myc-LRR domain immunoprecipitates incubated with cytochrome c, or not. A construct expressing a myc tagged NLRP3 LRR domain was transfected into HEK 293T cells. The Myc and control immunoprecipitates were incubated with cytochrome c (50 ng), washed, and immunoblotted. (D) Schematic of the constructs used in the above experiments (B & C). (E) Immunoblots of cell lysates and cardiolipin bead pull-downs to assess whether cytochrome c interferes with the interaction between cardiolipin and NLRP3. BSA (50 ng) or purified cytochrome c (50 ng) was added to lysates prepared from HEK 293T cells expressing NLRP3-Flag. Following a 30 minute incubation cardiolipin conjugated beads were added to the lysates for an additional 30 minutes. The caridolipin beads were washed; and the bound NLRP3 eluted in SDS-sample buffer, size fractionated by SDS PAGE, and quantitated by immunoblotting. The amount of NLRP3 in the cardiolipin pulldowns was normalized to the BSA control. The above experiments were respectively performed twice. (F) Cell lysates from cell treated with ATP, or not, were fractionated into cytosolic and mitochondrial fractions and the indicated proteins were immunoblotted. NLRP3 immunoprecipitates were prepared using the cytosolic fraction. An interaction between endogenous NLRP3 and cytochrome c was assess by immunoblotting. The ratios between the NLRP3 and cytochrome c levels in the immunoprecipitates were quantitated using Image J.

Next, we asked whether the released cytochrome c might target NLRP3 to either enhance or inhibit inflammasome activation. We first checked whether we could detect an interaction between endogenous NLRP3 and cytochrome c following inflammasome activation. We immunoprecipitated NLRP3 from THP-1 cell lysates prepared from LPS stimulated cells treated with ATP, or not, and immunoblotted for the presence of cytochrome c. A small amount of cytochrome c could be detected associated with NLRP3 even without ATP treatment, while a larger amount of cytochrome c co-immunoprecipitated with NLRP3 following ATP stimulation (Fig 2A). When normalized to the amount of NLRP3 immunoprecipitated we found approximately 3-fold more cytochrome c following ATP treatment. To map which domain of NLRP3 is needed to interact with cytochrome c, constructs expressing Myc tagged NLRP3 or truncated versions were transfected into HEK 293T cells. The expressed proteins were immunoprecipitation using antibodies to the Myc tag, washed, and incubated with a small amount of purified cytochrome c. After washing, the Myc immunoprecipitates were subjected to SDS-PAGE and immunoblotted for the Myc tag and cytochrome c. The results indicate that the NLRP3 LRR domain was necessary for binding cytochrome c (Fig 2B). We confirmed that the LRR domain was sufficient for this interaction by showing that an expressed NLRP3 LRR domain interacted with cytochrome c (Fig 2C). A schematic of full length NLRP3 and the various truncated versions are shown (Fig 2D). Because cardiolipin also binds to the LRR domain of NLPR3 [10], we tested whether cytochrome c and NLRP3 competed for cardiolipin binding. We prepared cell lysates from HEK 293T cells previously transfected with Flag tagged NLRP3. One set of lysates received cytochrome c and the other set BSA. Both set of lysates were incubated with cardiolipin conjugated beads and the interacting proteins precipitated, washed, fractionated by SDS-PAGE, transferred, and immunoblotted for NLRP3. Despite similar inputs of NLRP3 we pulled down less NLRP3 from the cytochrome c containing lysates than the BSA containing lysate (Fig 2E). This result is consistent with the binding of cytochrome c to the LRR repeats of NLRP3 reducing the interaction of NLRP3 with cardiolipin. However, another interpretation is that cytochrome c binds caridolipin hampering the interaction of NLRP3 with the cardiolipin beads. To verify whether cytochrome c interacts with NLRP3 in the cytosol, we treated THP-1 cells with LPS and ATP to release cytochrome c from mitochondria into cytosol. Cell lysates from these cells were separated into cytosolic and mitochondria enriched fractions and NLRP3 was immunoprecipitated from the cytosolic fraction. We then immunoblotted the immunoprecipitates for cytochrome c. Our data indicate that NLPR3 interacts with cytochrome c in the cytosol, and that ATP treatment releases more cytochrome c to interact with NLRP3 (Fig 2F).

### Cytochrome c inhibits NLRP3 inflammasome activation

**Fig 3:**
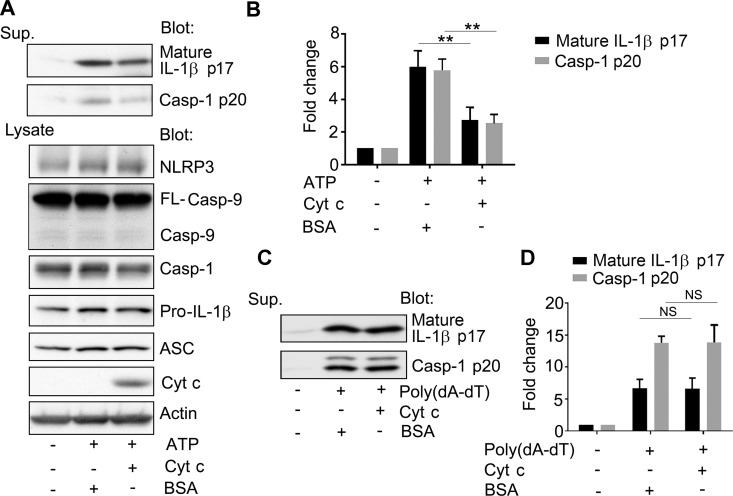
Increased cytosolic cytochrome c inhibits NLRP3, but not AIM2 inflammasome activation. (A) Immunoblots of cell lysates and supernatants for the expression of IL-1β, caspase-1, caspase-9, ASC, cytochrome c, and actin as indicated to assess the impact of cytochrome c on NLRP3 inflammasome activity. Cytochrome c or BSA was transduced into LPS-primed THP-1 cells for 3 hours, and during the last hour ATP was present in the cell culture. Cell lysates and supernatants were collected. Endogenous cytochrome c was visualized on longer exposure (not shown). (B) Quantification of the results from (A) and 2 other similar experiments. Results are shown as mean +/- SEM. The difference between the BSA and cytochrome c transduced cells were compared by Student *t* test using Prism software. ** indicates that p < 0.01. (C) Immunoblots of cell lysates for processed IL-1β and caspase-1 p20 to examine the impact of cytochrome c on AIM2 inflammasome activation. LPS primed (2 h) THP-1 cells were transduced with cytochrome c or BSA, and transfected with poly(dA-dT) for six hours. Supernatants were collected and analyzed. (D) Quantification of the results from (C) and 2 other similar experiments. Results are shown as mean +/- SEM. The difference between the BSA and cytochrome c transduced cells were compared by Student *t* test using Prism software. N.S. indicates non-significant. The above experiments were respectively performed 3 times.

To test whether cytosolic cytochrome c affects NLRP3 inflammasome activation, we delivered purified cytochrome c protein, or BSA as a control, using a lipid based protein transduction protocol. We found that the presence of cytochrome c reduced ATP-induced NLRP3 inflammasome activation as the levels of IL-1β and activated caspase-1 declined in the supernatant of THP-1 cells previously transduced with cytochrome c in comparison to those transduced with BSA (Fig 3A and 3B). Since the delivery of cytochrome c into the cytosol can also trigger the apoptosome by binding to Apaf-1, which will activate caspase-9 [1], we assessed whether the cytochrome c transduction protocol delivered sufficient cytochrome c to trigger casapase-9 cleavage. However, despite impairing NLRP3 inflammasome activity, we did not observe any significant change in the status of caspase-9 in the cell lysates of the cytochrome c transduced cells compared to the control cells (Fig 3A). Either the duration or the amount of cytochrome c transduced into the cell was insufficient to trigger detectable caspase-9 cleavage. Next, we checked whether cytoplasmic cytochrome c affected AIM2 inflammasome activation using a similar protein transduction protocol. We assessed IL-1β and caspase-1 levels in cell culture supernatants conditioned by LPS induced and Poly (dA/dT) transfected THP-1 cells that had been previously transduced with either cytochrome c or BSA. In contrast to the NLRP3 inflammasome activation we found no difference between the BSA and cytochrome c transduced cells (Fig 3C and 3D). These data argue that the release of low levels of cytochrome c during NLRP3 inflammasome activation may be sufficient to limit inflammasome activity and that the release of larger amounts of cytochrome c during apoptosis will likely inhibit NLRP3 inflammasome activity.

**Fig 4:**
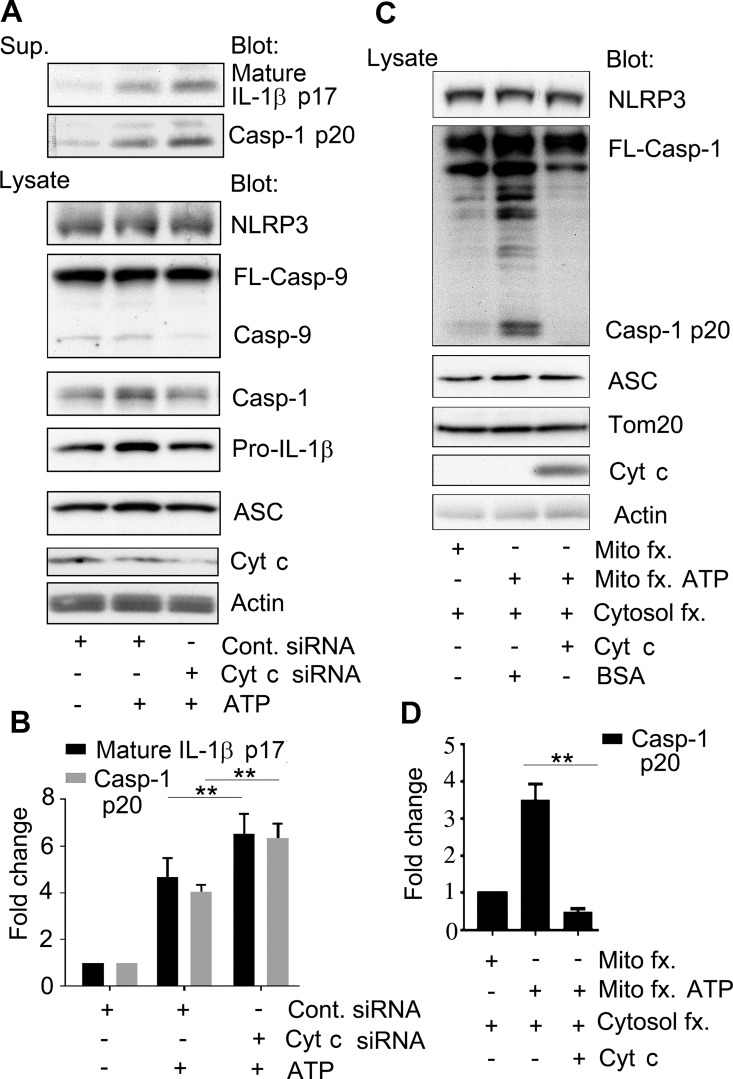
Reducing cytochrome c enhances NLRP3 inflammasome activation while exogenous cytochrome c inhibits the caspase-1 activation in a reconstitution assay. (A) Immunoblots of cell supernatants and cell lysates from LPS primed THP-1 previously transfected with cytochrome c or control siRNAs. The indicated proteins were immunoblotted. (B) Quantification of the results from (A) and 2 other similar experiments. Results are shown as mean +/- SEM of the fold increase of the indicated protein as assessed by Image J. The difference between the control and cytochrome c siRNA treated cells were compared by Student *t* test using Prism software. ** indicates that p < 0.01. (C) Immunoblot to assess caspase-1 activation *in vitro* using a cell fraction enriched for mitochondria and another for cytosolic proteins. The mitochondrial fraction was prepared from HEK 293T cells that had been treated with or without ATP. The cytosolic fraction was prepared from LPS primed THP-1 cells. The two fractions were mixed and incubated with either BSA or cytochrome c. The levels of the indicate proteins in the various mixtures are shown. (D) Quantification of the results from (C) and 2 other similar experiments. Results are shown as mean +/- SEM. The intensity of the bands determined using Image J. The differences in processed caspase-1 (p20) levels in the reactions containing BSA or cytochrome c were compared by Student *t* test using Prism software. ** indicates that p < 0.01. The above experiments were respectively performed 3 times.

To provide further evidence that cytochrome c modulates NLRP3 inflammasome activation, we reduced the expression of cytochrome c and then checked ATP-induced inflammasome activation. We found that silencing cytochrome c expression resulted in an increase in the amount of IL-1β and caspase-1 in the cell supernatant (Fig 4A and 4B). Since reducing cytochrome c could stress mitochondria releasing NLRP3 activators such as ROS, cardiolipin, or mtDNA contributing to the ATP-induced inflammasome activity we designed an *in vitro* caspase-1 assay to verify that cytochrome c can directly impair NLRP3 inflammasome induced caspase-1 activity. First, we treated THP-1 cells with LPS to provide a source of NLRP3, pro-IL1β, and pro-caspase-1. From these stimulated cells a cytosolic fraction was isolated. Second, HEK 293T cells, which lack significant amounts of NLRP3, ASC, and pro-caspase-1 [16, 17] were treated with ATP, or not, and then used to isolated a mitochondria enriched fraction. Next, we combined the two fractions, added BSA, and tested whether we could detect cleaved caspase-1 in the cell lysates (Fig 4C, lanes 1 and 2). Only the mitochondria fraction from the ATP treated cells was able to trigger capase-1 activity in the reconstituted cell lysate. Next, we replaced the BSA with cytochrome c. Now the ATP treated mitochondria fraction and the THP-1 cytosolic fraction failed to cleave caspase-1 (Fig 4C, lane 3). We checked the individual fractions for ASC expression and the mitochondria fractions for Tomm20, verifying equal loading and fraction integrity (Fig 4C). These data indicate that cytochrome c can interfere with NLRP3 activation.

### Cytochrome c reduces NEK7 induced NLRP3 inflammasome activation

**Fig 5:**
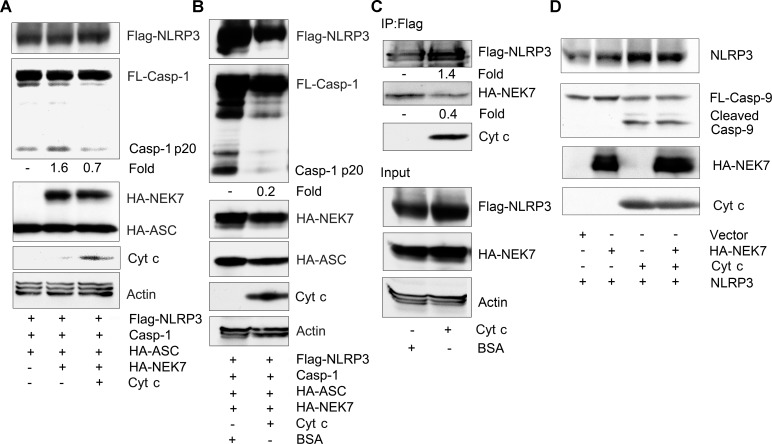
Cytochrome c reduces NEK7 mediated NLRP3 inflammasome activation and the interaction between NEK7 and NLRP3. (A) Immunoblots of cell lysates from HEK 293T cells transfected with constructs expressing FLAG-NLRP3, caspase-1, and HA-ASC in the presence or absence of HA-NEK7, and the presence or absences of construct expressing cytochrome c to assess caspase-1 cleavage. (B) Immunoblots of cell lysate from Flag-NLRP3, caspase-1, and HA-ASC transfected HEK 293T cells mixed with cell lysates from HA-NEK7 transfected HEK 293T cells and incubated with either BSA or cytochrome c to assess caspase-1 cleavage. The mixed cells lysates were incubated for 30 minutes at 30°C prior to immunoblotting for the indicated proteins. (C) Immunoblots of cell lysates and Flag-NLRP3 immunoprecipitates mixed with HA-NEK7 expressing cell lysates to assess whether cytochrome c affects the interaction between NLRP3 and NEK7. Cell lysates from HEK 293T cells expressing HA-NEK7 were added to Flag-NLRP3 immunoprecipitates and incubated with BSA or cytochrome c for 1 hour at room temperature. After which the anti-Flag beads were washed, the bound proteins eluted, and used for immunoblotting. Input levels in the cell lysates are also shown. (D) Immunoblots of a mixture of cell lysates prepared from LPS stimulated differentiated THP-1 cells and HEK 293T cell expressing HA-NEK7 to assess caspase-9 cleavage. The mixed cell lysates were incubated with cytochrome c (0.25 mg/ml) for 30 minutes at 30°C, or not. The above experiments were respectively performed twice.

Expression of NEK7 in conjunction with NLRP3 and ASC, and caspase-1 in HEK 293T cells was sufficient to trigger caspase-1 activation [9]. To test whether the presence of cytochrome c would also inhibit NEK7 induced caspase-1 activation we transfected the relevant proteins into HEK 293T cells and examined the cleavage of caspase-1. We found that in the absence of cytochrome c an increase in cleaved caspase-1 in NEK7 overexpressed cells, while co-expression of cytochrome c inhibited the increase (Fig 5A). To verify these results we did an *in vitro* reconstitution assay mixing cell lysates from cells expressing NLRP3, caspase-1, and ASC with lysates from NEK7 transfected cells. Incubation of the two lysates led to the cleavage of caspase-1 while the addition of recombinant cytochrome c substantially reduced the detection of cleaved caspase-1 (Fig 5B). To determine whether cytochrome c interfered with the interaction between NEK7 and NLRP3 we immunoprecipitated Flag-NLRP3 and added cell lysates from NEK7 transfectants in the presence of BSA or cytochrome c. Following a 1 hour incubation the Flag-NLRP3 immunoprecipitates were washed and immunoblotted for NEK7. We found a 70% reduction in the amount of NEK7 bound to the Flag-NLRP3 immunoprecipitates in the presence of cytochrome c suggesting that cytochrome c had interfered with the interaction between the two proteins (Fig 5C). Finally, we checked whether NEK7 had any effect on the cleavage of caspase-9 which it did not, nor did it affect the cleavage of caspase-9 triggered by the addition of cytochrome c (Fig 5D). However, this last assay was performed in vitro utilizing large amount of exogenously added cytochrome c. While these studies show that cytochrome c can interfere with the interaction between NEK7 and NLRP3 we had to rely on over expression studies to examine the interaction between NEK7 and NLRP3.

## Conclusions

The conceptual similarity between the NLRP3 inflammasome (NLR sensor, ASC, and caspase-1) and the apoptosome (Apaf-1, cytochrome c, and caspase-9) has been increasingly recognized [18, 19]. In response to specific activating signals both assemble multiprotein complexes that control inflammation and cell death. Mitochondrial damage and lysosomal membrane destabilization have been implicated in the activation of both of them [14, 20, 21]. There is also increasing evidence of cross-regulation between the signaling pathways that control their assembly. The inhibitors of apoptosis protein (IAPs), which are critical inhibitors of apoptotic signaling, have both positive and negative effects on inflammasome activation [22, 23]. In this study we confirmed the release of cytochrome c into the cytosol by signals that assemble NLRP3 inflammasomes. We found that cytochrome c interferes with the binding of NLRP3 to cardiolipin and NEK7. By introducing small amounts of cytochrome c into the cytosol by protein transduction we reduced NLRP3, but not AIM2 inflammasome activity. Conversely by reducing cytochrome c levels by gene silencing we enhanced IL-1β production following NLRP3 inflammasome activation. Using an *in vitro* caspase-1 activation assay that depended upon mitochondria purified from cells treated with ATP we found that the addition of exogenous cytochrome c markedly reduced the activation of caspase-1. Our findings contrast with experiments that showed a lack of involvement of cytochrome c in NALP-1 (NLRP1) inflammasome activation [24]. Human NLRP1 features an amino-terminal PYD, a NOD, LRRs, a function-to-find domain and a carboxy-terminal CARD domain. Although NLRP1 and NLRP3 both have LRR repeats, NLRP3 has nine repeats, while NLRP1 has only six. Alignments of their LRR repeats show approximately 50% identity. This suggests that the LRR repeats of NLRP3 and NALP1 differ such that cytochrome can interact with NLRP3, but not with NLRP1. Following apoptotic signals the release of large amounts of cytochrome c likely limits the concurrent activation of NLRP3 inflammasomes. Further study of the interactions between NEK7, cardiolipin and cytochrome c with NLRP3 may provide a means to modulate NLRP3 inflammasome activity.

## References

[1] Wang X. The expanding role of mitochondria in apoptosis. Genes Dev. 2001;15(22):2922–33. Epub 2001/11/17. 11711427

[2] Fink SL, Cookson BT. Apoptosis, pyroptosis, and necrosis: mechanistic description of dead and dying eukaryotic cells. Infect Immun. 2005;73(4):1907–16. Epub 2005/03/24. 73/4/1907 [pii]. 10.1128/IAI.73.4.1907-1916.2005 15784530 PMC1087413

[3] Lamkanfi M, Dixit VM. Mechanisms and Functions of Inflammasomes. Cell. 2014;157(5):1013–22. Epub 2014/05/27. S0092-8674(14)00475-9 [pii]. 10.1016/j.cell.2014.04.007 24855941

[4] Lamkanfi M, Dixit VM. Inflammasomes and their roles in health and disease. Annu Rev Cell Dev Biol. 2012;28:137–61. Epub 2012/09/15. 10.1146/annurev-cellbio-101011-155745 22974247

[5] Py BF, Kim MS, Vakifahmetoglu-Norberg H, Yuan J. Deubiquitination of NLRP3 by BRCC3 critically regulates inflammasome activity. Mol Cell. 2013;49(2):331–8. 10.1016/j.molcel.2012.11.009 23246432

[6] Hara H, Tsuchiya K, Kawamura I, Fang R, Hernandez-Cuellar E, Shen Y, et al Phosphorylation of the adaptor ASC acts as a molecular switch that controls the formation of speck-like aggregates and inflammasome activity. Nat Immunol. 2013;14(12):1247–55. 10.1038/ni.2749 24185614 PMC4813763

[7] Shi H, Wang Y, Li X, Zhan X, Tang M, Fina M, et al NLRP3 activation and mitosis are mutually exclusive events coordinated by NEK7, a new inflammasome component. Nat Immunol. 2016;17(3):250–8. PubMed Central PMCID: PMC4862588. 10.1038/ni.3333 26642356 PMC4862588

[8] Schmid-Burgk JL, Chauhan D, Schmidt T, Ebert TS, Reinhardt J, Endl E, et al A Genome-wide CRISPR (Clustered Regularly Interspaced Short Palindromic Repeats) Screen Identifies NEK7 as an Essential Component of NLRP3 Inflammasome Activation. J Biol Chem. 2016;291(1):103–9. PubMed Central PMCID: PMC4697147. 10.1074/jbc.C115.700492 26553871 PMC4697147

[9] He Y, Zeng MY, Yang D, Motro B, Nunez G. NEK7 is an essential mediator of NLRP3 activation downstream of potassium efflux. Nature. 2016;530(7590):354–7. PubMed Central PMCID: PMC4810788. 10.1038/nature16959 26814970 PMC4810788

[10] Iyer SS, He Q, Janczy JR, Elliott EI, Zhong Z, Olivier AK, et al Mitochondrial cardiolipin is required for Nlrp3 inflammasome activation. Immunity. 2013;39(2):311–23. Epub 2013/08/21. S1074-7613(13)00329-4 [pii]. 10.1016/j.immuni.2013.08.001 23954133 PMC3779285

[11] Gonzalvez F, Gottlieb E. Cardiolipin: setting the beat of apoptosis. Apoptosis. 2007;12(5):877–85. 10.1007/s10495-007-0718-8 17294083

[12] Gonzalvez F, Schug ZT, Houtkooper RH, MacKenzie ED, Brooks DG, Wanders RJ, et al Cardiolipin provides an essential activating platform for caspase-8 on mitochondria. J Cell Biol. 2008;183(4):681–96. Epub 2008/11/13. jcb.200803129 [pii]. 10.1083/jcb.200803129 19001123 PMC2582890

[13] Nakahira K, Haspel JA, Rathinam VA, Lee SJ, Dolinay T, Lam HC, et al Autophagy proteins regulate innate immune responses by inhibiting the release of mitochondrial DNA mediated by the NALP3 inflammasome. Nat Immunol. 2011;12(3):222–30. Epub 2010/12/15. ni.1980 [pii]. 10.1038/ni.1980 21151103 PMC3079381

[14] Shimada K, Crother TR, Karlin J, Dagvadorj J, Chiba N, Chen S, et al Oxidized mitochondrial DNA activates the NLRP3 inflammasome during apoptosis. Immunity. 2012;36(3):401–14. Epub 2012/02/22. S1074-7613(12)00044-1 [pii]. 10.1016/j.immuni.2012.01.009 22342844 PMC3312986

[15] Germain M, Mathai JP, Shore GC. BH-3-only BIK functions at the endoplasmic reticulum to stimulate cytochrome c release from mitochondria. J Biol Chem. 2002;277(20):18053–60. 10.1074/jbc.M201235200 11884414

[16] Bryan NB, Dorfleutner A, Rojanasakul Y, Stehlik C. Activation of inflammasomes requires intracellular redistribution of the apoptotic speck-like protein containing a caspase recruitment domain. J Immunol. 2009;182(5):3173–82. PubMed Central PMCID: PMCPMC2652671. 10.4049/jimmunol.0802367 19234215 PMC2652671

[17] Ichinohe T, Yamazaki T, Koshiba T, Yanagi Y. Mitochondrial protein mitofusin 2 is required for NLRP3 inflammasome activation after RNA virus infection. Proc Natl Acad Sci U S A. 2013;110(44):17963–8. PubMed Central PMCID: PMCPMC3816452. 10.1073/pnas.1312571110 24127597 PMC3816452

[18] Sutterwala FS, Haasken S, Cassel SL. Mechanism of NLRP3 inflammasome activation. Ann N Y Acad Sci. 2014;2014. Epub 2014/05/21. 10.1111/nyas.12458 PMC4074217 24840700

[19] Riedl SJ, Salvesen GS. The apoptosome: signalling platform of cell death. Nat Rev Mol Cell Biol. 2007;8(5):405–13. Epub 2007/03/23. nrm2153 [pii]. 10.1038/nrm2153 17377525

[20] Zhou R, Yazdi AS, Menu P, Tschopp J. A role for mitochondria in NLRP3 inflammasome activation. Nature. 2011;469(7329):221–5. Epub 2010/12/03. nature09663 [pii]. 10.1038/nature09663 21124315

[21] Hornung V, Bauernfeind F, Halle A, Samstad EO, Kono H, Rock KL, et al Silica crystals and aluminum salts activate the NALP3 inflammasome through phagosomal destabilization. Nat Immunol. 2008;9(8):847–56. PubMed Central PMCID: PMCPMC2834784. 10.1038/ni.1631 18604214 PMC2834784

[22] Labbe K, McIntire CR, Doiron K, Leblanc PM, Saleh M. Cellular inhibitors of apoptosis proteins cIAP1 and cIAP2 are required for efficient caspase-1 activation by the inflammasome. Immunity. 2011;35(6):897–907. 10.1016/j.immuni.2011.10.016 22195745

[23] Vince JE, Wong WW, Gentle I, Lawlor KE, Allam R, O'Reilly L, et al Inhibitor of apoptosis proteins limit RIP3 kinase-dependent interleukin-1 activation. Immunity. 2012;36(2):215–27. 10.1016/j.immuni.2012.01.012 22365665

[24] Martinon F, Burns K, Tschopp J. The inflammasome: a molecular platform triggering activation of inflammatory caspases and processing of proIL-beta. Mol Cell. 2002;10(2):417–26. 12191486 10.1016/s1097-2765(02)00599-3

